# Pharmacological Modulation of Dopamine Receptor D2-Mediated Transmission Alters the Metabolic Phenotype of Diet Induced Obese and Diet Resistant C57Bl6 Mice

**DOI:** 10.1155/2011/928523

**Published:** 2011-04-06

**Authors:** J. E. de Leeuw van Weenen, E. T. Parlevliet, J. P. Schröder-van der Elst, S. A. van den Berg, K. Willems van Dijk, J. A. Romijn, H. Pijl

**Affiliations:** ^1^Department of Endocrinology and Metabolic Diseases, Leiden University Medical Center, P.O. Box 9600, 2300 RC Leiden, The Netherlands; ^2^Department of Human Genetics, Leiden University Medical Center, P.O. Box 9600, 2300 RC Leiden, The Netherlands

## Abstract

High fat feeding induces a variety of obese and lean phenotypes in inbred rodents. Compared to Diet Resistant (DR) rodents, Diet Induced Obese (DIO) rodents are insulin resistant and have a reduced dopamine receptor D2 (DRD2) mediated tone. We hypothesized that this differing dopaminergic tone contributes to the distinct metabolic profiles of these animals. C57Bl6 mice were classified as DIO or DR based on their weight gain during 10 weeks of high fat feeding. Subsequently DIO mice were treated with the DRD2 agonist bromocriptine and DR mice with the DRD2 antagonist haloperidol for 2 weeks. Compared to DR mice, the bodyweight of DIO mice was higher and their insulin sensitivity decreased. Haloperidol treatment reduced the voluntary activity and energy expenditure of DR mice and induced insulin resistance in these mice. Conversely, bromocriptine treatment tended to reduce bodyweight and voluntary activity, and reinforce insulin action in DIO mice. These results show that DRD2 activation partly redirects high fat diet induced metabolic anomalies in obesity-prone mice. Conversely, blocking DRD2 induces an adverse metabolic profile in mice that are inherently resistant to the deleterious effects of high fat food. This suggests that dopaminergic neurotransmission is involved in the control of metabolic phenotype.

## 1. Introduction

Dopamine is intimately involved in the regulation of energy balance. Genetically engineered dopamine-deficient mice fail to initiate feeding and consequently die of starvation, unless L-DOPA, the precursor of dopamine, is provided daily [1]. Conversely, dopamine release in response to food intake induces satiety and reward [2]. Thus, dopamine plays an important dual role in the complex physiology driving meal initiation and termination. Moreover, dopaminergic neurotransmission profoundly affects glucose and lipid metabolism [3].

Dopamine action is mediated by 5 distinct G-protein coupled receptor subtypes, functionally classified into 2 receptor families according to their effect on target neurons. Activation of dopamine receptor D2 (DRD2), D3, or D4, comprising the D2 family, inhibits adenylyl cyclase. Activation of the receptors belonging to the D1 family (DRD1 and DRD5) stimulates adenylyl cyclase [4].

Dopaminergic transmission is altered in insulin resistant and obese animals. Basal and feeding-evoked dopamine release is exaggerated in several nuclei of the hypothalamus of obese Zucker rats [5–7], whereas DRD2 expression is reduced in hypothalamic nuclei of obese animal models [8, 9]. The number of DRD2 binding sites in the striatum of obese humans is reduced and inversely correlated with body mass index [10].

Modulation of DRD2 activity profoundly affects energy homeostasis in humans and animals. Drugs that block DRD2 enhance appetite and induce weight gain in animals and humans [11–14]. Conversely, DRD2 agonist drugs reduce body weight, increase energy expenditure, and improve glycemic control in obese animals and individuals [15–18]. 

High fat feeding induces obesity, insulin resistance, and diabetes in rodents. However, the amount of weight gained in response to a high fat diet varies considerably, even among animals with a genetically identical background [19–21]. Indeed, diet sensitive (diet induced obese, DIO) rodents display several alterations in pathways regulating energy homeostasis compared to diet resistant (DR) rodents [21, 22], and DIO and DR rodents differ with respect to various components of their dopaminergic system, even before the onset of obesity [23, 24]. In particular, DIO mice and rats are characterized by an increased expression of dopamine transporter and reduced DRD2 expression [23]. In view of the evidence summarized above, altered DRD2-mediated neurotransmission could contribute to the metabolic phenotype of these animals. We hypothesized that modulation of dopaminergic transmission in DIO and DR mice with DRD2 agonist or antagonist drugs would redirect the metabolic phenotypes of these mice. We particularly postulated that stimulation of DRD2 would ameliorate insulin resistance of DIO C57Bl6 mice, whereas DRD2 antagonism would induce insulin resistance in DR animals of the same strain. To address this hypothesis, DIO and DR mice were treated with bromocriptine, a DRD2 agonist, or haloperidol, a DRD2 antagonist, respectively. After 1 week of treatment, energy metabolism was measured in a Comprehensive Laboratory Animal Monitoring System, and after 2 weeks a hyperinsulinemic euglycemic clamp was performed to quantify insulin action, in particular, with respect to its propensity to inhibit lipolysis.

## 2. Experimental Procedure

### 2.1. Animals

Seventy-two male C57Bl6Jico mice, 11 or 12 weeks old, (Charles River, Maastricht, The Netherlands) were housed in a temperature- and humidity-controlled room on a 12-h light-dark cycle with free access to food and water, unless mentioned otherwise. All animal experiments were performed in accordance with the principles of laboratory animal care and regulations of Dutch law on animal welfare, and the experimental protocol was approved by the Animal Ethics Committee of the Leiden University Medical Center.

### 2.2. Experimental Design

All mice were maintained on a high fat diet (45 energy% of fat derived from palm oil, 35 energy% of carbohydrate, and 20 energy% of protein—Research Diet Services, Wijk bij Duurstede, The Netherlands). After 10 weeks of high fat feeding, the 24 mice with the highest weight gain were classified as DIO mice and the 24 mice with the lowest weight gain were classified as DR mice. The 24 mice with intermediate weight gain were not further used in this study.

DIO and DR mice were randomly divided into a placebo and treatment group. DR treated mice received haloperidol (1 mg/kg/day), DIO treated mice received bromocriptine (10 mg/kg/day), and DIO and DR placebo mice received placebo treatment. Subcutaneous implantable haloperidol, bromocriptine, and placebo pellets (Innovative Research of America, Florida, USA), ensuring continuous release of the medication were used to treat mice. Pellets were implanted under isoflurane anesthesia. Mice were treated for 2 weeks, meanwhile maintained on the high fat diet.

### 2.3. Measurement of Energy Metabolism

Mice were subjected to indirect calorimetric measurements for a period of 3 consecutive days using a Comprehensive Laboratory Animal Monitoring System (CLAMS; Columbus Instruments, Ohio, USA). Due to a limited number of cages, eight mice per group were measured. Mice were allowed to acclimatize to the cages for a period of 14 hours prior to the start of the experiment. Measurements started at 7:00 am and continued for 72 hours. The CLAMS system enables real-time continuous monitoring of food intake, drinking behavior, activity, and metabolic gas exchange. Oxygen consumption (VO_2_) and carbon dioxide production rates (VCO_2_) were measured at 7 minute intervals. The respiratory exchange rate (RER), as a measure for metabolic substrate choice, was calculated using the following formula:

(1) $$\begin{array}{c} \text{RER}=\frac{{\text{VCO}}_{\text{2}}}{{\text{VO}}_{\text{2}}}. \end{array}$$

Carbohydrate and fat oxidation rates were calculated from VO_2_ and VCO_2_ using the following formulas [25]:

(2) $$\begin{array}{c} \text{Carbohydrate}\;\text{oxidation}\left(\text{kcal}/\text{h}\right)=\frac{\left(\left(4.585*{\text{VCO}}_{2}\right)-\left(3.226*{\text{VO}}_{2}\right)\right)*4}{1000},\\ \text{Fat}\;\text{oxidation}\;\left(\text{kcal}/\text{h}\right)=\frac{\left(\left(1.695*{\text{VO}}_{2}\right)-\left(1.701*{\text{VCO}}_{2}\right)\right)*9}{1000}. \end{array}$$

VO_2_ and VCO_2_ are in mL/h.

Total energy expenditure was calculated as the sum of carbohydrate and fat oxidation. Activity was monitored by infrared beam breaks across the *x*- and *y*-axis. All energy metabolism data was calculated separately for day and night time.

### 2.4. DEXAScan

Body composition was measured by dual-energy X-ray absorptiometry (DEXA) using the Norland pDEXA Sabre X-Ray Bone Densitometer (Norland, Hampshire, UK). Before measuring, mice were anesthetized with a combination of 6.25 mg/kg acepromazine (Alfasan, Woerden, The Netherlands), 6.25 mg/kg midazolam (Roche, Mijdrecht, The Netherlands) and 0.3125 mg/kg fentanyl (Janssen-Cilag, Tilburg, The Netherlands).

### 2.5. Hyperinsulinemic Euglycemic Clamp

Prior to the experiment, mice were fasted for 16 hours after food withdrawal at 5:00 pm. Hyperinsulinemic euglycemic clamp studies started at 9:00 am and were performed as described earlier [26]. During the experiment, mice were anesthetized with a combination of 6.25 mg/kg acepromazine (Alfasan, Woerden, The Netherlands), 6.25 mg/kg midazolam (Roche, Mijdrecht, The Netherlands) and 0.3125 mg/kg fentanyl (Janssen-Cilag, Tilburg, The Netherlands). First, basal rate of glycerol turnover was determined by giving a primed (0.6 *μ*Ci) continuous (0.9 *μ*Ci/h) intravenous (i.v.) infusion of [1-(3)-^3^H]-Glycerol (GE Healthcare, Little Chalfont, UK) for 60 minutes. Subsequently, insulin (Novo Nordisk, Denmark) was administered in a primed (4.5 mU) continuous (6.8 mU/h) i.v. infusion for 90 minutes to attain a steady state circulating insulin concentration of ~6 *μ*g/L.

Every 10 min the plasma glucose concentration was determined via tail vein bleeding (<3 *μ*l) (Accu-chek, Sensor Comfort, Roche Diagnostics GmbH, Mannheim, Germany) and accordingly the i.v. infusion rate of a 12.5% D-glucose solution was adjusted to maintain euglycemia. Blood samples (60 *μ*L) were taken during the basal period (at 50 and 60 min) and during the hyperinsulinemic period (at 70, 80, and 90 min) to determine plasma concentrations of glucose, insulin, nonesterified fatty acids (NEFA), free glycerol, and ^3^H-Glycerol specific activities. At the end of the clamp, mice were sacrificed.

### 2.6. Analytical Procedures

Commercially available kits were used to determine the plasma concentration of glucose (Instruchemie, Delfzijl, The Netherlands), NEFA (Wako, Nuess, Germany), and free glycerol (Sigma, MO, USA). The plasma insulin concentration was measured by an ELISA (Mercodia AB, Uppsala, Sweden). Total plasma ^3^H-Glycerol was determined in plasma and in supernatant after trichloroacetic acid (20%) precipitation and water evaporation.

### 2.7. Calculations

The turnover rate of glycerol (*μ*mol/min/kg) was calculated during the basal period and under steady-state hyperinsulinemic conditions as the rate of tracer infusion (dpm/min) divided by the plasma-specific activity of ^3^H-Glycerol (dpm/*μ*mol). The turnover rates were corrected for body weight.

### 2.8. Statistical Analysis

Data is presented as mean ± standard deviation. Statistical analysis was performed using SPSS. A one-way ANOVA was used for analysis of the data. If significant differences were found, the LSD method was applied as posthoc test to determine differences between 2 groups. Statistical differences were only shown when apparent between DIO and DR placebo groups, between DIO placebo and bromocriptine groups, or between DR placebo and haloperidol groups. Differences were considered statistically significant when *P* < .05.

## 3. Results

### 3.1. Body Weight and Basal Plasma Metabolites

**Figure 1:**
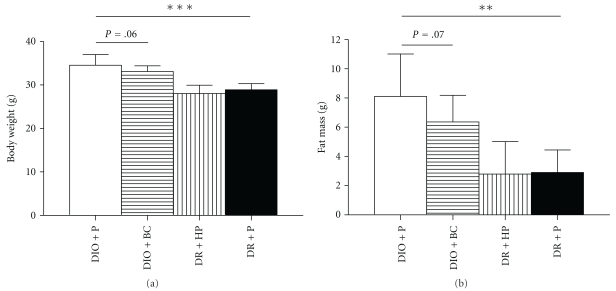
Bodyweight (a) and fat mass (b) of DIO and DR mice after treatment with bromocriptine (BC), haloperidol (HP), or placebo (P) for 2 weeks. Data is presented as mean ± SD for 12 (a) or 10 (b) mice per group. **P < .01; ***P < .001.

Mice were designated DIO or DR according to their weight gain following a 10-week high fat diet. By definition, DIO mice had a significantly higher bodyweight compared to DR mice after this dietary pretreatment (35.4 ± 1.5 versus 30.6 ± 1.9; *P* < .001), which was completely accounted for by a difference in fat mass (Figure 1(b)). Lean body mass did not differ (not shown). Two weeks of placebo treatment did not alter the difference in bodyweight between DIO and DR mice (Figure 1(a)). Two weeks of bromocriptine treatment tended to induce weight loss in DIO mice (primarily fat mass, Figure 1(b)), although the effect did not reach statistical significance. Haloperidol did not impact on the bodyweight of DR mice.

**Figure 2:**
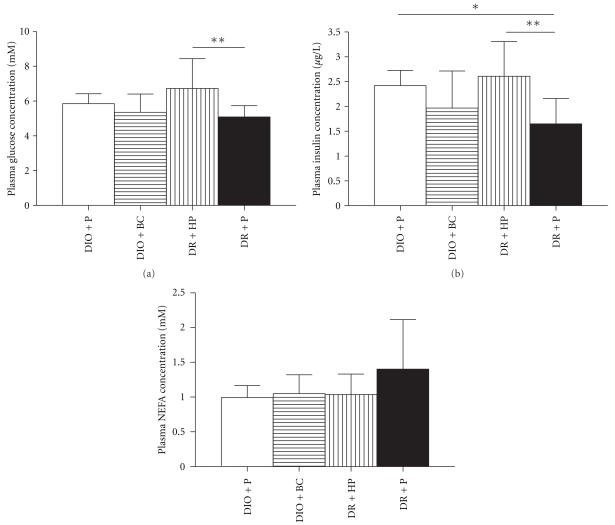
Fasting plasma glucose (a), insulin (b), and NEFA (c) concentrations in DIO and DR mice after treatment with bromocriptine (BC), haloperidol (HP), or placebo (P) for 2 weeks. Data is presented as mean ± SD for 9 or 10 mice per group. *P < .05; **P < .01.

The fasting plasma glucose concentration was not different between placebo treated DIO and DR mice (Figure 2(a)), whereas the fasting plasma insulin concentration was significantly elevated in DIO mice (Figure 2(b)). Haloperidol significantly increased fasting plasma glucose and insulin concentrations in DR mice, while the insulin and glucose concentrations in DIO mice remained unchanged upon bromocriptine treatment. The fasting plasma NEFA concentration did not differ between the groups (Figure 2(c)).

### 3.2. Energy Metabolism

**Figure 3:**
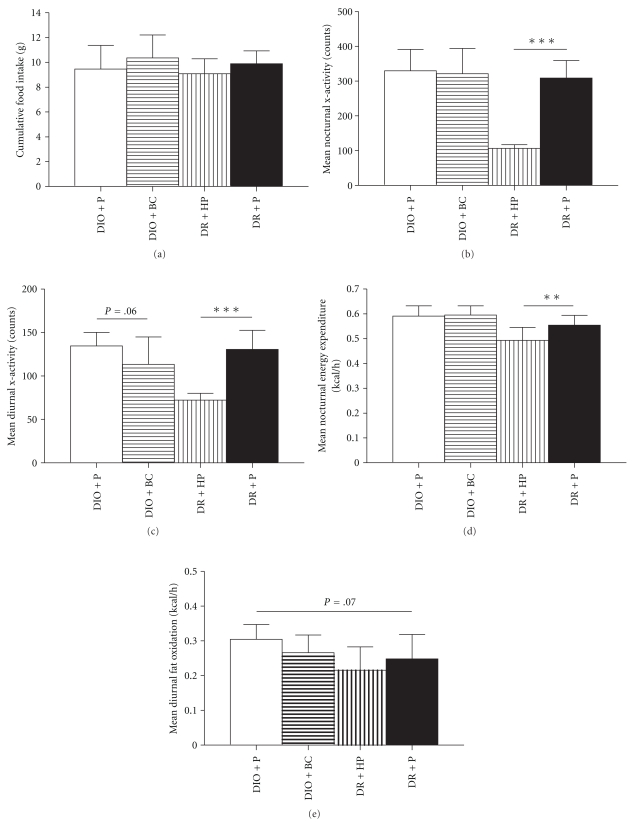
Cumulative food intake (a), mean nocturnal (b) and diurnal *x*-axis activity (c), mean nocturnal energy expenditure (d), and mean diurnal fat oxidation rate (e) in DIO and DR mice after treatment with bromocriptine (BC), haloperidol (HP), or placebo (P) for 1 week. Data is presented as mean ± SD for 7 or 8 mice per group. **P < .01; ***P < .001.

After 1 week of treatment, whole body energy metabolism of mice was assessed with a Comprehensive Laboratory Animal Monitoring System using indirect calorimetry. Individual food intake, activity, and respiratory gas exchange was monitored for 3 consecutive days. Cumulative food intake (Figure 3(a)), voluntary activity (Figures 3(b) and 3(c)), energy expenditure (Figure 3(d)) as well as the carbohydrate oxidation rate (data not shown) did not differ between placebo treated DIO and DR mice. The diurnal fat oxidation rate tended to be higher in DIO mice, but this failed to reach statistical significance (Figure 3(e)). Diurnal and nocturnal voluntary activity in DR mice was dramatically reduced by haloperidol (Figures 3(b) and 3(c)), and this was accompanied by a reduction in whole body nocturnal energy expenditure (Figure 3(d)). The impact of haloperidol on fat (Figure 3(e)) and carbohydrate oxidation (data not shown) did not reach statistical significance. Food intake was not affected by haloperidol treatment (Figure 3(a)). The diurnal voluntary activity tended to be lower in DIO mice receiving bromocriptine, but this also failed to reach statistical significance (Figure 3(c)). Furthermore, bromocriptine treatment had no significant effect on food intake (Figure 3(a)), energy expenditure (Figure 3(d)), fat oxidation (Figure 3(e)), or carbohydrate oxidation rate (data not shown).

### 3.3. Insulin Action

After 2 weeks of treatment, mice were subjected to a hyperinsulinemic euglycemic clamp. Basal and hyperinsulinemic plasma glucose, insulin, free glycerol, and NEFA concentrations are shown in Table 1. The plasma NEFA concentration was reduced to the same extent in all groups during hyperinsulinemia.

**Figure 4:**
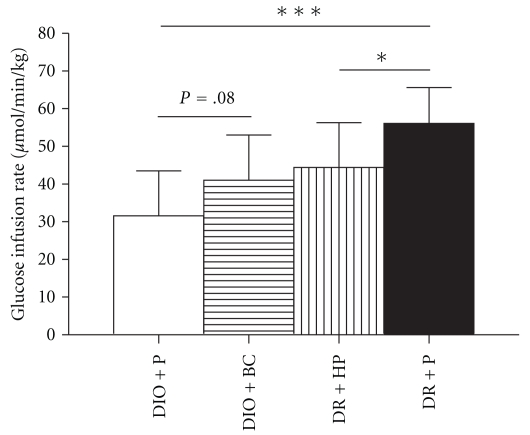
Glucose infusion rate during a hyperinsulinemic euglycemic clamp in DIO and DR mice after treatment with bromocriptine (BC), haloperidol (HP), or placebo (P) for 2 weeks. Data is presented as mean ± SD for 9 or 10 mice per group. *P < .05; ***P < .001.

The glucose infusion rate necessary to maintain euglycemia was significantly higher in DR compared to DIO mice (Figure 4), which indicates that DIO mice were insulin resistant compared to DR animals. Haloperidol significantly diminished the glucose infusion rate in DR mice, reflecting a deterioration of insulin action, whereas bromocriptine tended to increase glucose infusion required to maintain euglycemia in DIO mice (indicating improved insulin action). The capacity of insulin to inhibit glycerol turnover was not different between DR and DIO mice, and it was not affected by either drug (data not shown).

## 4. Discussion

The results presented here demonstrate that pharmacological modulation of dopaminergic transmission by a DRD2 agonist or antagonist can partly redirect the divergent metabolic phenotypes of DIO and DR mice. In particular, blocking dopaminergic transmission by means of haloperidol induces insulin resistance of glucose metabolism in DR mice. Conversely, activation of dopaminergic neurotransmission by bromocriptine tended to ameliorate insulin resistance in DIO animals. These data suggest that DRD2-mediated neurotransmission is involved in the control of glucose and insulin metabolism.

Although they have a genetically identical background, individual C57Bl6 mice show distinct susceptibility to develop obesity and insulin resistance when maintained on a high fat diet. We classified mice as DIO or DR based on the amount of weight gained during 10 weeks of high fat feeding. DIO mice were insulin resistant compared to DR mice, as evidenced by higher fasting plasma insulin levels and lower glucose infusion rate required to maintain euglycemia during insulin infusion. These findings are in accordance with other rodent studies [19–21, 24, 27]. Remarkably, there was no measurable difference in food intake, energy expenditure, or voluntary physical activity in DIO compared to DR mice.

DIO mice have significantly lower DRD2 expression levels in certain brain areas compared to DR mice [23]. Also, dopamine turnover is reduced in hypothalamic nuclei of DIO rats even before the onset of obesity [24], and the hypothalamus is intimately involved in the control of glucose and lipid metabolism [28, 29]. Since pharmacological activation of DRD2 ameliorates insulin resistance, in various obese animal models [17, 30], we hypothesized that modulation of DRD2-mediated neurotransmission could reverse the metabolic phenotypes of DIO and DR mice. In keeping with this hypothesis, blocking DRD2 by haloperidol induced insulin resistance in DR mice, whereas activation of DRD2 by bromocriptine tended to improve insulin sensitivity in DIO mice. In concert, these data suggest that DRD2 activation is involved in the control of glucose metabolism and that reduced dopaminergic transmission via DRD2 contributes to the metabolic phenotype (insulin resistance) of obese animals. However, we cannot exclude the possibility that the observed effects of bromocriptine and haloperidol are (partly) mediated by receptors other than DRD2. Haloperidol is also known to have a high affinity for DRD3, DRD4 and adrenergic *α*1 receptors [31], and bromocriptine also possesses high affinity for DRD3, the serotonergic 5-HT1A and 1D receptors, and the adrenergic *α*1 and *α*2 receptors [32]. Each of these receptors might participate in the impact of haloperidol and/or bromocriptine on energy and nutrient homeostasis. Adrenergic receptors (AR) are involved in the control of energy expenditure and glucose metabolism. Stimulation of *α*2-AR reduces spontaneous physical activity [33] and impairs insulin secretion [34–36]. Accordingly, overexpression of *α*2A-AR is associated with glucose intolerance [37]. Stimulation of *α*1-AR, on the other hand, has a positive impact on glucose homeostasis by promoting glucose uptake by adipose and muscle tissue [38–40] and absence of the *α*1B-AR leads to hyperinsulinemia and insulin resistance [41]. Acute stimulation of the 5-HT1A receptor increases food intake [42, 43], reduces plasma insulin levels and induces a concomitant rise in plasma glucose levels [44, 45]. As far as we know, the specific impact of DRD3, DRD4, and 5-HT1D receptors on the regulation of energy and nutrient homeostasis is still unknown. Thus, the effects of bromocriptine and haloperidol we observe here may be the ultimate result of modulation of various of these receptor activities. 

The fact that haloperidol induced insulin resistance is consistent with literature reporting an increased incidence of diabetes among individuals treated with haloperidol [46]. Interestingly, treatment with haloperidol is not associated with (massive) weight gain in humans [47], which also fits with our data and suggests that the drug hampers insulin action via mechanistic routes other than obesity. First, haloperidol dramatically reduced physical activity of DR mice. This is in agreement with a wealth of data from other animal experiments [48, 49]. Diminished locomotor activity hampers insulin action in muscle [50, 51]. Second, a major (side) effect of haloperidol treatment is elevation of prolactin levels [52, 53] which may contribute to the induction of glucose intolerance and insulin resistance [54, 55]. Third, haloperidol may alter glucose metabolism by modifying plasma levels of peptide hormones. The data documenting effects of haloperidol on leptin levels are inconsistent; increased [56] as well as unchanged leptin levels in response to haloperidol treatment have been reported [57, 58]. Furthermore, haloperidol seems to increase plasma ghrelin levels, while leaving levels of adiponectin, resistin, and visfatin unaffected [56]. Both leptin and ghrelin may impact insulin sensitivity directly [59, 60]. Fourth, haloperidol may diminish glucose-induced insulin secretion by blocking D2 receptors on pancreatic *β*-cells [61, 62], which leads to (postprandial) hyperglycemia. In the long run, hyperglycemia diminishes insulin action through “toxic” effects on insulin sensitive tissues [63]. Fifth, blockade of central DRD2 may induce insulin resistance via modulation of autonomic nervous output to peripheral tissues (including muscle, adipose tissue, and liver) [64].

Bromocriptine treatment tended to improve insulin sensitivity of glucose metabolism in DIO animals, but its effect on glucose infusion rate did not reach statistical significance. It is important to note that the route of administration of bromocriptine we used here may have diminished the efficacy of the drug. Indeed, it has been shown that subcutaneous, compared to intraperitoneal, administration of the drug limits its metabolic impact [65]. The tendency we observed though is in line with data obtained in diet induced obese hamsters [66], and genetically engineered obese mice [67]. In accordance, short-term administration of bromocriptine ameliorates various metabolic anomalies in obese humans without affecting body weight [18] and longer term treatment improves glycemic control and serum lipid profiles in patients with type 2 diabetes [68]. In addition, DRD2 agonists improve glucose and lipid metabolism in patients with hyperprolactinemia [69, 70] and acromegaly [71–73]. Although DRD2 agonists generally benefit nutrient metabolism, the use of these drugs is sometimes associated with the development of impulse control disorders, including binge and compulsive eating, in patients with Parkinson's disease, which may lead to excessive weight gain and insulin resistance [74, 75].

The effects of bromocriptine on metabolism may be mediated by central dopamine receptors, as is suggested by Luo et al. [17] who showed that intracerebroventricular administration of low dose bromocriptine during 14 days improves insulin sensitivity in obese, insulin-resistant, hamsters. However, peripheral receptors might also be involved. We previously reported that bromocriptine acutely impairs insulin secretion by stimulating the *α*2-AR on *β*-cells [36]. To explain that (sub)chronic bromocriptine treatment improves glucose metabolism [15, 66, 76], we hypothesized that suppression of insulin secretion induces *β*-cell “rest”, which might allow *β*-cells to replenish insulin stores, thereby enhancing the secretory capacity in the long run [77, 78]. It might also increase the number of organ-specific insulin receptors leading to improved insulin sensitivity [79, 80]. In addition, bromocriptine may alter glucose metabolism via modulation of circulating peptide levels. In obese women, bromocriptine reduces leptin concentrations [81]; the biological relevance of this for the results reported by us is questionable however, as leptin *improves* insulin sensitivity [59]. The impact of bromocriptine on other regulatory peptide hormones remains to be determined.

In summary, activation of DRD2 tends to ameliorate the metabolic profile of DIO mice, whereas antagonism of these receptors induces insulin resistance in DR mice. In concert with previous findings by other groups indicating that dopaminergic (DRD2 mediated) neurotransmission is reduced in the brain of DIO mice, our data suggest that DRD2-mediated dopaminergic mechanisms may be involved in the development of the divergent metabolic phenotypes in response to high fat feeding in C57Bl6 mice.

## Figures and Tables

**Table 1:** Plasma glucose, insulin, free glycerol, and NEFA concentrations during the basal and hyperinsulinemic conditions of the hyperinsulinemic euglycemic clamp in DIO and DR mice after treatment with bromocriptine, haloperidol, or placebo for 2 weeks.

	Clamp condition	DIO mice		DR mice	
		Placebo	Bromocriptine	Placebo	Haloperidol
Glucose (mM)	Basal	5.9 ± 0.6	5.3 ± 1.1	5.0 ± 0.6	6.7 ± 1.7
	Hyperinsulinemia	5.4 ± 0.7	5.6 ± 0.6	6.0 ± 0.7	4.8 ± 1.1
Insulin (*μ*g/L)	Basal	2.4 ± 0.3	2.0 ± 0.8	1.6 ± 0.5	2.6 ± 0.7
	Hyperinsulinemia	6.8 ± 1.9	7.2 ± 1.4	6.7 ± 1.3	7.1 ± 0.6
Free glycerol (mM)	Basal	0.1 ± 0.1	0.1 ± 0.1	0.2 ± 0.1	0.2 ± 0.1
	Hyperinsulinemia	0.1 ± 0.0	0.1 ± 0.0	0.1 ± 0.0	0.1 ± 0.0
NEFA (mM)	Basal	1.0 ± 0.2	1.1 ± 0.3	1.1 ± 0.2	1.0 ± 0.3
	Hyperinsulinemia	0.5 ± 0.1	0.5 ± 0.1	0.5 ± 0.2	0.5 ± 0.1

Data is measured in 9 or 10 mice and presented as mean ± SD.

## Abbreviations

AR: Adrenergic receptor
DIO: Diet induced obese
DR: Diet resistant
DRD2: Dopamine receptor D2
NEFA: Nonesterified fatty acids.
